# Salivary mycobiome dysbiosis and its potential impact on bacteriome shifts and host immunity in oral lichen planus

**DOI:** 10.1038/s41368-019-0045-2

**Published:** 2019-07-02

**Authors:** Yan Li, Kun Wang, Bo Zhang, Qichao Tu, Yufei Yao, Bomiao Cui, Biao Ren, Jinzhi He, Xin Shen, Joy D. Van Nostrand, Jizhong Zhou, Wenyuan Shi, Liying Xiao, Changqing Lu, Xuedong Zhou

**Affiliations:** 1 State Key Laboratory of Oral Diseases, National Clinical Research Center for Oral Diseases, West China Hospital of Stomatology, Sichuan University, 610041 Chengdu, China; 2 Institute of Marine Science and Technology, Shandong University, 266237 Qingdao, China; 3 Institute for Environmental Genomics, Department of Microbiology and Plant Biology, University of Oklahoma, Norman, OK 73019 USA; 4 The Forsyth Institute, Cambridge, MA 02142 USA; 5 Department of Anatomy, West China School of Basic Medical and Forensic Medicine, Sichuan University, 610041 Chengdu, China

**Keywords:** High-throughput screening, Epidemiology, High-throughput screening, Epidemiology

## Abstract

The biodiversity of the mycobiome, an important component of the oral microbial community, and the roles of fungal–bacterial and fungal–immune system interactions in the pathogenesis of oral lichen planus (OLP) remain largely uncharacterized. In this study, we sequenced the salivary mycobiome and bacteriome associated with OLP. First, we described the dysbiosis of the microbiome in OLP patients, which exhibits lower levels of fungi and higher levels of bacteria. Significantly higher abundances of the fungi *Candida* and *Aspergillus* in patients with reticular OLP and of *Alternaria* and *Sclerotiniaceae_unidentified* in patients with erosive OLP were observed compared to the healthy controls. *Aspergillus* was identified as an “OLP-associated” fungus because of its detection at a higher frequency than in the healthy controls. Second, the co-occurrence patterns of the salivary mycobiome–bacteriome demonstrated negative associations between specific fungal and bacterial taxa identified in the healthy controls, which diminished in the reticular OLP group and even became positive in the erosive OLP group. Moreover, the oral cavities of OLP patients were colonized by dysbiotic oral flora with lower ecological network complexity and decreased fungal–Firmicutes and increased fungal–Bacteroidetes sub-networks. Third, several keystone fungal genera (*Bovista*, *Erysiphe*, *Psathyrella*, etc.) demonstrated significant correlations with clinical scores and IL-17 levels. Thus, we established that fungal dysbiosis is associated with the aggravation of OLP. Fungal dysbiosis could alter the salivary bacteriome or may reflect a direct effect of host immunity, which participates in OLP pathogenesis.

## Oral Diseases: Role of fungal interaction in oral lichen planus

Imbalance in the oral fungal community could lead to the development of oral lichen planus (OLP), a chronic inflammatory disease that affects the mucous membranes in the mouth. The exact cause of OLP is uncertain, which is a major obstacle to therapeutic development. Using salivary samples, a team headed by Xuedong Zhou at Sichuan University in China investigated the composition and diversity of the fungal community in OLP patients and healthy individuals. The authors found that the oral fungal community was less diverse and that there were higher levels of bacteria in OLP patients. The team concluded that fungal community imbalance could affect the bacterial community in the saliva and the host immunity in the mucous membrane, thereby constituting a direct or indirect cause of the development of OLP.

## Introduction

Oral lichen planus (OLP) is a chronic oral mucosal disease that occurs in approximately 0.5%–2% of the general adult population,^1^^,2^ with an even higher prevalence among women. In clinical settings, OLP is classified into three subtypes (reticular, atrophic, and ulcerative) and affects the buccal mucosa in the vast majority of cases. The gingiva, tongue, and lips may also be affected. The reticular form is typically asymptomatic and is the most common type, characterized by the presence of Wickham striae. However, the atrophic and erosive types may cause different degrees of discomfort and soreness, demonstrating high risks for malignant transformation at rates of 1%–2% (a range of 0%–12.5%).^3,4^

The precise aetiology of OLP is uncertain, which is a major obstacle in the development of new therapeutics. Various factors have been considered to be potential causes of OLP, such as infection, immunity, genetic factors, stress, and trauma.^2^ However, the precise roles of these factors have been debated. Over the last decade, microbial infection has received increasing attention in the context of OLP pathogenesis. Previously, we evaluated differences in the salivary microbial communitites between OLP patients and healthy individuals. We observed that the bacterial community in saliva from OLP patients was characterized by greater variety and less bacterial specificity, comprising only *Porphyromonas* and *Solobacterium*,^1^ which exhibited significantly higher abundances compared with the healthy controls. Additionally, a decrease in *Streptococcus* and an increase in gingivitis/periodontitis-associated bacteria were observed in OLP lesions in another study.^5^ These findings implicated a link between oral bacterial dysbiosis and OLP. It is noteworthy that the oral cavity is colonized by both bacteria and fungi, the latter of which have been known to have a role in OLP for a long time. Among oral fungi, *Candida* species have been reported to be associated with OLP and are detected in 37%–50% of OLP patients.^6^ *Candida albicans* is the most predominant OLP-associated *Candida* species and is involved in the malignant transformation of OLP.^7^ The carriage rate for *C. albicans* in patients with erosive OLP is much higher than that observed in patients with non-erosive OLP or in healthy controls.^8^ Additionally, non-*C. albicans* species have been specifically isolated from OLP patients, indicating a possible association between these yeasts and OLP.^9^ However, previous studies have primarily focused on fungal epidemiology, such as the carriage rate for *Candida* species in OLP patients, with few studies analysing the biodiversity and composition of the entire fungal community living in symbiosis with bacteria in the oral ecosystem. The advent of the use of next-generation sequencing technology to evaluate microbial diversity has broadened our view of the importance of fungi. The salivary mycobiome, which primarily refers to the fungal microbiota, is an important component of the oral microbiome. Ghannoum et al.^10^ and Dupuy et al.^11^ evaluated the complexity of the core oral mycobiomes of healthy individuals. However, these findings did not significantly contribute to a wider understanding of the disease state. Moreover, the interaction between the mycobiome and the resident bacterial microbiome may be crucial for the progression of diseases such as inflammatory bowel disease, cystic fibrosis, and oral diseases.^12–14^ Interactions between fungi and commensal bacteria involve physical binding, signalling molecule communication, and metabolic exchange in oral niches.^14^ Although microbial infection has been proposed to be a causative, associated, or possibly worsening factor in OLP, little is known regarding the oral fungal–bacterial relationship in OLP progression.

In addition, OLP is considered a T cell-mediated inflammatory disease because the infiltrating lymphocytes are primarily T cells. Recently, the Th17 subset of CD4+ T helper cells was shown to play a crucial role in promoting immune inflammatory reactions in the defence against infection by extracellular microorganisms and in autoimmune disease. Moreover, numerous Th17 cells have been identified in OLP lesions,^15^ and interleukin (IL)-17 and IL-23, cytokines secreted by Th17 cells, are important components involved in the defence against pathogenic microorganisms.^16^ For example, salivary IL-17 and IL-23 are significantly correlated with specific bacterial genera in OLP, such as *Porphyromonas*, indicating their potential roles in the pathogenic mechanism of OLP.^4^ Accumulating evidence has also implicated IL-17 and IL-23 in immunity to fungal pathogens, with the IL-23/IL-17 axis being essential in the defence against *Pneumocystis carinii*. Conversely, this axis amplifies the inflammatory pathology in mouse models of *Candida* or *Aspergillus* infection.^17^

Similar to the gut microbiome,^12,18,19^ inter-kingdom interactions between bacteria and fungi may be substantial in the oral cavity. Because the host immune system is a major stress that modulates microbial composition,^14,20–22^ perturbations in salivary IL-17 and IL-23 levels, as well as the altered oral bacteriome observed in our previous study, suggests a disequilibrium within the oral mycobiomes of OLP patients. To test this hypothesis, we evaluated the salivary fungal abundance, frequency, and diversity in OLP and explored the complex and dynamic ecological relationships between the fungal mycobiome, oral bacteria, and host immunity. Our results indicated that fungal community composition and diversity are dramatically altered among OLP patients. Thus, despite their numerical inferiority, the oral mycobiome may be a driving force for bacteriome shifts though the modulation of mucosal immunity, which directly or indirectly affects OLP pathogenicity.

## Results

### Participant demographics and sequence data

The subjects enrolled in this study included 18 healthy subjects (age (39.72 ± 11.02) years), 17 reticular OLP patients (age (43.58 ± 9.97) years), and 18 erosive OLP patients (age (46.72 ± 9.80) years). There were no significant differences in the age and gender distributions among the groups (*P* = 0.127 and *P* = 0.815 respectively). The severity of OLP was scored using a semiquantitative scoring system based on the site, area, and clinical presence of lesions.^23^

Using an Illumina MiSeq sequencing platform, 1 580 028 raw paired-end reads of ITS region amplicons were obtained. After merging the forward and reverse reads and performing quality trimming, 712 295 merged sequences with an average length of 324 bp were obtained for all 53 samples. These sequences (335 185 for the healthy control samples, 197 963 for the reticular OLP samples, and 179 147 for the erosive OLP samples) were then clustered into 4 564 OTUs after quality trimming, dereplication, clustering, and chimera removal using the UPARSE pipeline, with an OTU identity cutoff of 97%. Of the 4 564 identified OTUs, 1 990 were singletons. The taxonomic assignments made using the RDP classifier showed that 4 563 OTUs were fungi, with only 1 OTU with 2 sequences identified as protozoan but with 20% confidence. Among the fungal ITS OTUs, 1 588 belonged to Ascomycota, 20 to Chytridiomycota, 976 to Basidiomycota, 52 to Zygomycota, and 15 to Glomeromycota. The remaining OTUs were either assigned to unidentified fungi (124 OTUs) or known fungal phyla with <50% confidence (1 789 OTUs).

### Lower saliva biodiversity of the mycobiome and higher biodiversity of the bacteriome in OLP

**Fig. 1:**
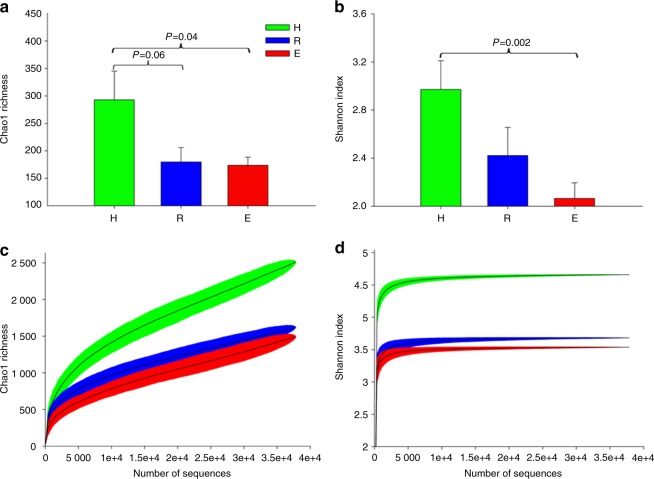
Diversity analysis of the salivary fungal communities in the healthy subject (H), reticular OLP (R), and erosive OLP (E) groups. The fungal community of erosive OLP displayed significantly lower richness and α-diversity compared to the healthy controls for various diversity measures (*P* < 0.05). **a** Chao1 richness. **b** Shannon index. **c** Rarefaction curves of Chao1 richness obtained by combining samples in the same group. **d** Rarefaction curves of the Shannon index obtained by combining samples in the same group

We estimated the community diversity for all of the samples to compare their complexity among reticular OLP, erosive OLP, and healthy individuals. Significantly lower richness and alpha diversity were observed for the fungal communities in the erosive OLP group compared with the healthy subjects (*P* < 0.05; Fig. 1a, b). The same trend was also observed between the reticular OLP and healthy control samples, but no significant difference was detected (*P* > 0.05; Fig. 1a, b). Rarefaction analyses indicated that fungal species richness and alpha diversity among the three groups gradually decreased as the disease was aggravated (Fig. 1c, d), which was in contrast to the tendency of the bacteriome (Table S1).^1^

The phylogenetic structure was further analysed. Although unweighted principal coordinate analysis showed no obvious separation among the mycobiomes of the healthy subjects, reticular OLP and erosive OLP (Fig. S1), dissimilarity tests, including MRPP, adonis and ANOSIM, did reveal significant differences between the healthy control group and the two OLP groups (*P* < 0.05; Table 1). However, no dramatic differences were detected in reticular OLP when it was compared with erosive OLP (*P* > 0.05; Table 1).

**Table 1:** Comparison of the overall fungal community structure using three non-parametric statistical methods

Items	MRPP		adonis		ANOSIM	
	*δ*	*P*	*F*	*P*	*R*	*P*
H vs R	0.777	0.001	0.083	0.002	0.144	0.005
H vs E	0.807	0.002	0.079	0.002	0.171	0.001
R vs E	0.725	0.254	0.038	0.16	0.02	0.162

H, healthy control; R, reticular OLP; E, erosive OLP

### Taxonomic differences among healthy individuals and OLP patients

**Fig. 2:**
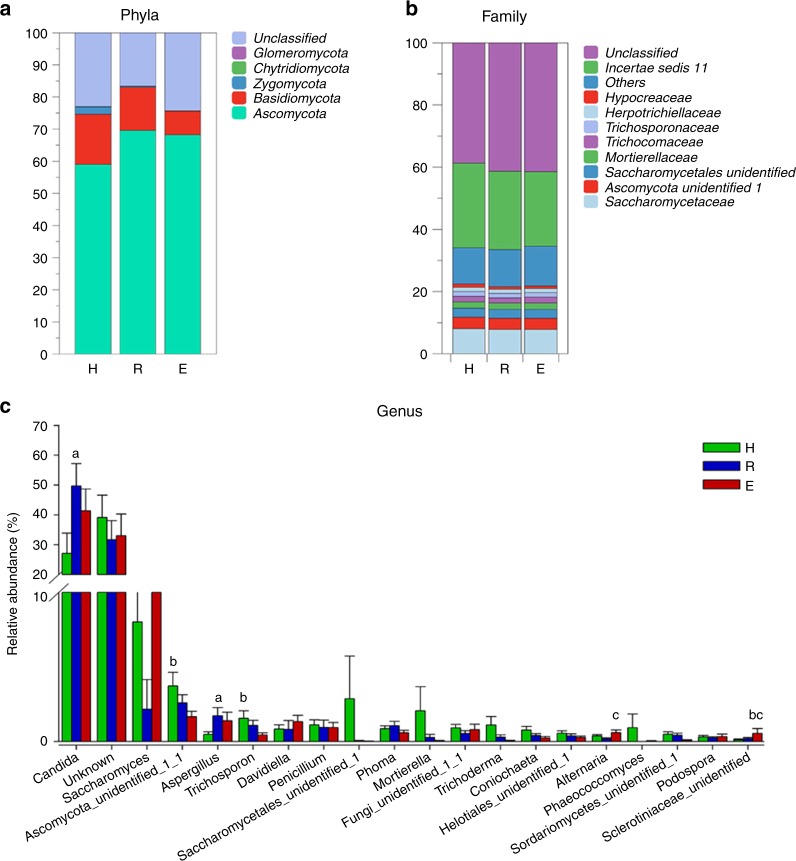
Relative abundances of fungal phyla, families and predominant genera (*P* > 0.1%) among the healthy subject (H), reticular OLP (R), and erosive OLP (E) groups. **a** Phylum level. **b** Family level. **c** Comparison of the top 20 abundant genera. **a** H vs R; **b** H vs E; **c** R vs E; superscript letters indicate *P* < 0.05

The fungal community composition was analysed at different taxonomic levels. At the phylum level, significantly different patterns were observed for the top two prevalent phyla: *Ascomycota* (59.03% in healthy individuals, 69.58% in reticular OLP, and 68.22% in erosive OLP) and *Basidiomycota* (15.62%, 13.46%, and 7.33%, respectively; Fig. 2a). The phylum *Ascomycota* showed higher abundance in the reticular and erosive OLP groups, whereas the abundance of *Basidiomycota* was lower in the OLP groups compared with the healthy controls. At the family level, there were 11 fungal families for which no significant difference was observed between the OLP patients and healthy individuals (Fig. 2b).

At the genus level, a total of 280 genera were detected. Among them, 126 genera were only present in one individual. The abundances of several genera were significantly different among the groups (Fig. 2c). The relative abundances of *Candida* and *Aspergillus* were significantly increased in the reticular OLP group compared with those observed in the healthy subjects. In contrast, *Ascomycota_unidentified_1_1* and *Trichosporon* were strikingly more abundant in the healthy subjects than in those with erosive OLP. Furthermore, significantly higher levels of *Alternaria* and *Sclerotiniaceae_unidentified* were observed in the erosive OLP group compared with the reticular OLP group.

**Fig. 3:**
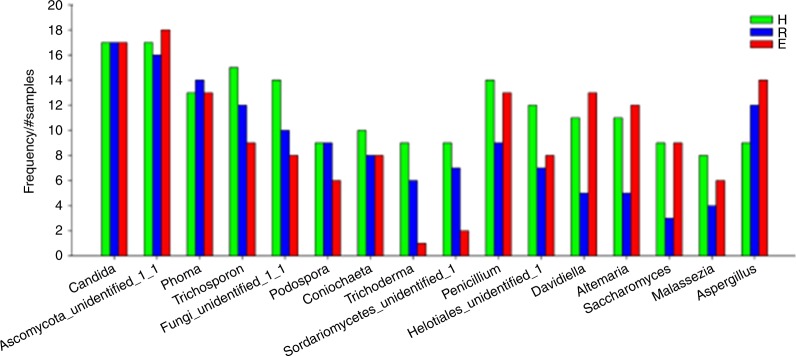
Frequency of fungal genera with average relative abundance above 0.1% among the healthy subject (H), reticular OLP (R), and erosive OLP (E) groups. A total of 16 genera were included in this analysis

The most frequently detected fungi (constituting the “core” mycobiome) at the genus level with an average relative abundance above 0.1% are shown in Fig. 3. Among them, *Candida* and *Ascomycota_unidentified_1_1* were the two genera with the highest detectable frequencies (96%) in all three groups. In addition, the frequencies of *Phoma*, *Trichosporon*, *Penicillium*, *Aspergillus*, *Fungi_unidentified_1_1*, and *Coniochaeta* were above 50% in all of the samples. No “OLP-specific” taxa (present in either the healthy or OLP groups) were detected. However, we identified *Aspergillus* as an “OLP-associated” fungus, as it was present at a higher frequency in the OLP group than in healthy controls.

To further investigate the key oral fungal microbiota associated with OLP, we evaluated the genera and OTUs with frequencies of at least 50% and relative abundances of ≥0.5%. *Aspergillus* was only present in the reticular OLP group, while *Phoma* was detected in both the healthy subject and reticular OLP groups (Fig. S2a). Although *Candida* and *Ascomycota_unidentified_1_1* were detected in all three groups, *Candida* was more abundant in the reticular OLP group, and the abundance of *Ascomycota_unidentified_1_1* was significantly increased in the healthy subjects (Fig. 2c). Additionally, in terms of OTU levels, we observed that OTU_4 429 (*Candida*) and OTU_21 (*Phoma*) were only present in the two OLP groups and were absent in the healthy control group (Fig. S2b).

### Inversion of myco-bacteriome co-occurrence patterns from antagonization to co-prosperity

**Fig. 4:**
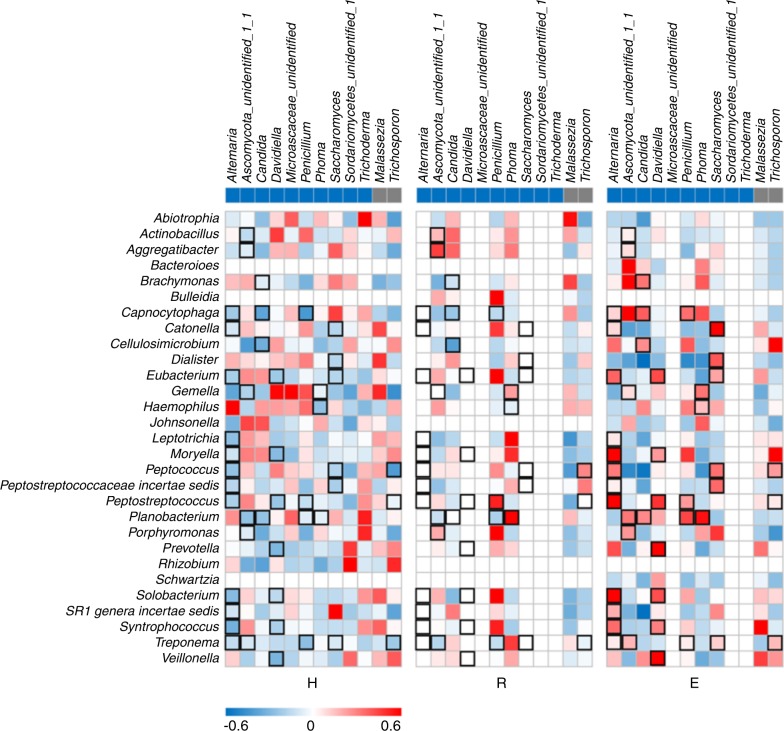
Co-occurrence relationships between abundant fungal and bacterial genera across samples. Co-occurrence and co-exclusion relationships of genera present in at least eight subjects were explored by Pearson correlation coefficient analysis. The bacterial genera are shown on the left, and the fungal genera are positioned at the top. Fungal genera belonging to *Ascomycota* are marked in blue, while *Basidiomycota* genera are marked in grey. Rectangle frames are used to highlight the negative myco-bacteriome co-occurrence interactions in the healthy control group, which changed to positive in the erosive OLP group

Given the observation that bacterial–fungal interactions are actively present throughout the human body and that certain fungal taxa are distinctly distributed, we hypothesized that bacteriome–mycobiome co-occurrence and co-exclusion networks differed between the OLP patients and healthy controls. The bacterial–fungal network was constructed by only including the genera detected in no fewer than eight subjects. In total, 12 fungal and 29 bacterial genera were included, as shown in Fig. 4. Several interesting findings were obtained from the network. First, among the healthy individuals, most of the myco-bacteriome co-occurrence interactions were negative, whereas positive co-occurrence relationships were observed in the erosive OLP group. However, in the reticular OLP group, half of the correlations disappeared because some of the enrolled fungi were not detected in this group. Second, as the predominant fungal genus, *Candida* exhibited 12 significant inversions (negative to positive) with bacterial genera, of which six genera were identified in the reticular OLP group (*Abiotrophia*, *Actinobacillus*, *Aggregatibacter*, *Dialister*, *SR1genera incertae sedis*, and *Treponema*) and six were observed in the erosive OLP group (*Bacteroides*, *Brachymonas*, *Capnocytophaga*, *Cellulosimicrobium*, *Planobacterium*, and *Veillonella)* (Table S2).

### Distinct network topology between OLP and healthy individuals

**Fig. 5:**
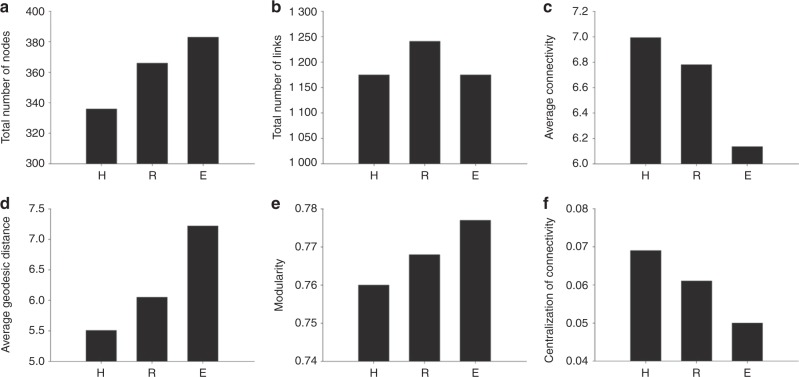
Fungal–bacterial co-occurrence network analysis of the healthy subject (H), reticular OLP (R), and erosive OLP (E) groups. Various network indices were used to describe the properties of the fungal–bacterial co-occurrence patterns, including the total number of nodes **a**, total number of links **b**, average connectivity **c**, average geodesic distance **d**, modularity **e**, and centralization of connectivity **f**

**Fig. 6:**
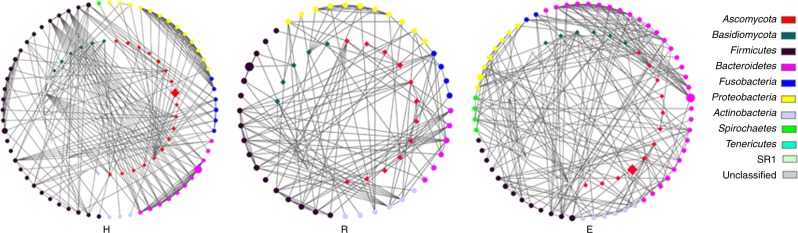
Sub-network analysis of fungal–bacterial relationships in the healthy subject (H), reticular OLP (R), and erosive OLP (E) groups. Sub-networks for the H, R, and E groups were constructed by extracting all of the bacterial OTUs connected with the fungal OTUs. The nodes in the inner circle are fungal OTUs and nodes in the outer circle are bacterial OTUs

We also constructed co-occurrence ecological networks at the OTU level by incorporating both fungal and bacterial OTUs to predict their ecological relationships involved in OLP (Fig. S3). Strikingly, the co-occurrence or mutual exclusion patterns of the three groups were significantly different. Decreased network complexity was observed from the healthy to the erosive OLP stages. In total, 1 175 associations and 336 nodes were observed in the healthy control group network, 1 241 associations and 366 nodes were observed in the reticular OLP network, and 1 175 associations and 383 nodes were observed in the erosive OLP network. The constructed healthy control group network showed an average connectivity of 6.994, an average geodesic distance of 5.509, a modularity of 0.76 and a centralization of connectivity value of 0.069, while the networks of reticular and erosive OLP had average connectivities of 6.781 and 6.136, average geodesic distances of 6.05 and 7.22, modularities of 0.768 and 0.777, and centralization of connectivity values of 0.061 and 0.05, respectively (Fig. 5; Table S3). This finding was further confirmed via sub-networks constructed by extracting the first bacterial neighbours of the fungal nodes with the highest connectivity (Fig. 6). Several interesting findings were observed. The number of significant correlations involving members of the phylum Firmicutes (black nodes, the majority belonging to *Streptococcus*) clearly decreased in the erosive OLP network compared with the healthy control network. In contrast, the involvement of OTUs from the phylum Bacteroidetes (rose-red nodes, primarily *Prevotella*, *Porphyromonas*, and *Capnocytophaga*) in the co-occurrence network increased significantly in the erosive OLP network. For fungal genera belonging to the phylum *Ascomycota*, such as *Candida*, far fewer co-occurrence events were observed in the reticular OLP network than in the erosive OLP network.

### Fungal disturbance promotes OLP exacerbation

**Fig. 7:**
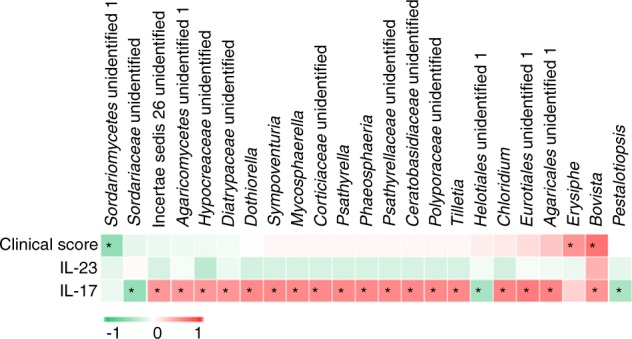
Relationship between the relative abundances of fungal genera and clinical parameters. Three clinical parameters were analysed, including the clinical score and IL-17 and IL-23 levels. Pearson correlation coefficient was performed. * indicates *P* < 0.05

We also examined the relationship between fungal genera and clinical parameters based on Pearson correlation coefficient values. The salivary concentrations of IL-17 and IL-23 were measured using an enzyme-linked immunosorbent assay (ELISA).^1^ In total, 29 fungal genera were observed to have significant correlations with clinical parameters, including clinical scores and salivary levels of IL-17 and IL-23, and were therefore identified as keystone fungi in saliva (Fig. 7). Several interesting correlations were observed. First, there were significant positive correlation patterns between clinical scores and the fungal genera *Erysiphe* and *Bovista*, whereas *Sordariomycetes unidentified 1* was negatively correlated with clinical scores. Second, regarding the correlation with immunologic factors involved in the inflammatory response in OLP, several fungal genera, such as *Dothiorella*, *Sympoventuria*, and *Mycosphaerella*, showed significant positive correlations with salivary levels of IL-17. However, *Sordariaceae unidentified*, *Helotiales unidentified 1,* and *Pestalotiopsis* were negatively correlated with IL-17. Notably, no significant correlation was observed between salivary levels of IL-23 and fungal genera. Finally, among the fungal genera associated with clinical data, significant correlations with more than one parameter were determined for genera such as *Bovista*, which positively correlated with clinical scores and salivary levels of IL-17 simultaneously.

## Discussion

Although numerous studies have emphasized the possible role of bacterial or viral infection in OLP,^1,4^ the fungal component of the oral microbiome has not been thoroughly investigated. In the present study, we showed for the first time the structural characteristics of the core mycobiome in salivary samples from reticular and erosive OLP patients, which demonstrated lower biodiversity and an increased abundances and frequencies of the genera *Candida* and *Aspergillus*.

The oral fungal community was less enriched in OLP patients compared with that observed in the healthy control group. Interestingly, the opposite pattern was observed for the bacteriome, which demonstrated significantly increased diversity in the OLP group compared to the healthy control group. The fungi-to-bacteria diversity ratio decreased sharply in the OLP group compared to the healthy control group. OLP is quite different from most other mucocutaneous diseases, such as atopic dermatitis, psoriasis, Crohn’s disease, and ulcerative colitis, which are associated with decreased diversity of the bacteriome^1,24–26^ and an increased diversity of the mycobiome.^19^ This inverted mycobiome-to-bacteriome trend was similar to the results obtained by Hoarau in the gastrointestinal tract.^18^ The results of a previous study showed that the *Candida* load negatively correlates with salivary bacterial diversity.^27^ In addition, a study by Peleg et al.^28^ showed that anaerobic bacteria otherwise inhibit fungi. Specific alterations in fungal diversity in parallel with variations in bacterial diversity implicate an oral micro-ecological imbalance in OLP.

Previous studies^10,11^ have reported that more than 100 fungal species are members of the oral flora. The results of our study further demonstrated the existence oral mycobiota diversity, identifying 6 phyla, 11 families, and 280 genera of fungi. In particular, we evaluated the patterns of fungal genera associated with OLP, demonstrating an increase in opportunistic/pathogenic fungi and a decrease in symbiotic fungi. The relative abundance of *Candida* was higher in the reticular and erosive OLP groups (49.6% and 41.3%, respectively) than in the healthy subject group (27.1%), although a significant difference was only observed between the reticular OLP and healthy control groups. This result was in complete accordance with previous findings.^29^ We propose the following possible causes of this increase in *Candida* abundance. First, the susceptibility of OLP patients to *Candida* may be increased compared with healthy controls. Second, *Candida* hyphae may prefer the nonlesional reticular mucosa to erosive mucosa. Third, the types of pathogenic *Candida* in the saliva of OLP patients may be different from those in healthy individuals, a hypothesis that is supported by our analyses at the OTU level. OTU_3 662 (*Candida*) dominated in the saliva of the healthy control group, while the core species in the reticular and erosive OLP groups was OTU_4 429 (*Candida*). Hoarau et al.^18^ showed that *C. tropicalis* rather than *C. albicans* is the pathogen responsible for Crohn’s disease. *Aspergillus*, another opportunistic fungal pathogen involved in endodontic infection, cystic fibrosis,^30,31^ and immunocompromised patients, may cause a spectrum of respiratory disease, wound infections and biofilm formation on medical devices. We also observed a significantly higher abundance and frequency of *Aspergillus* in OLP patients than in the healthy control group. In cases of oral lesions associated with dimorphic fungi (*Candida*), filamentous fungi (*Aspergillus* spp.) have been reported to be present, but these instances typically involve severe immunosuppression and disseminated infection to extraoral sites.^32^ Taking these findings into consideration, it is possible that alterations in the fungal population are driven by an expansion of *Candida* and *Aspergillus* in the oral mycobiota of OLP individuals. Similar results have revealed a higher susceptibility to *Candida* and *Aspergillus* infection in the absence of Toll IL1R8 (TIR8), a negative regulator of Th17 responses.^33^ The overgrowth of native *Candida* and *Aspergillus* species may be positively correlated with OLP severity, suggesting a disease link. Moreover, a fungal genus associated with invasive diseases, *Alternaria*, was observed to have a richer abundance in individuals with erosive OLP rather than reticular OLP, indicating its potential pathogenicity with the development of OLP. Artico et al.^34^ showed that asthma severity is associated with the presence of *Alternaria* species in the lung that may have originally been derived from the mouth. Significant differences in the abundance of Sclerotiniaceae, which has also been detected in Crohn’s disease,^35^ were observed between the erosive OLP group compared to reticular OLP and healthy control groups, possibly because it is a family of necrotrophic fungi. The results of the studies referenced above indicate that the oral mycobiome is involved in specific oral diseases as well as in respiratory and digestive diseases.

Sixteen genera were present with frequencies greater than 20% in each group and were designated the “core” mycobiome, which exhibited substantial overlap with the core oral mycobiota described in two previous studies. Specifically, our results are in good agreement with those of Ghannoum et al.^10^ and Dupuy et al.^11^ with respect to the identification of *Candida*, *Alternaria*, *Aspergillus*, *Cladosporium/Davidella*, *Saccharomyces*, *Phoma*, and *Malassezia*. However, nine oral cavity-associated genera were uniquely identified in our study, including *Ascomycota_unidentified_1_1*, *Trichosporon*, and *Fungi_unidentified_1_1*, and *Podospora*, among others. *Candida* species were the most prevalent in both healthy and diseased oral cavities, demonstrating a 96% carriage rate in the samples assayed in our study, higher than that observed in other studies (60%–80%)^1,10^ and much higher than the culture rate of 17.7%.^32^

In addition to analysing disease-associated fungi, we further confirmed significant shifts in the salivary fungal–bacterial interactions in OLP patients by exploring the differences in microbial co-occurrence and co-exclusion patterns between healthy and OLP individuals. The most dominant fungal genus, *Candida*, was of particular interest. *Candida* was negatively correlated with 18 out of 29 bacterial genera in healthy individuals. In contrast, *Candida* was positively correlated with eight bacterial genera in reticular OLP and eight bacterial genera in erosive OLP. Some of them (*Treponema*, *Aggregatibacter*, *Dialister*, *SR1*, *Bacteroides*, *Capnocytophaga*, and *Veilonella*) are strict anaerobic periodontopathogenic genera. How such strict anaerobes survive in an aerobic niche such as the oral cavity may be explained by the relationship between *Candida* and the high level of O_2_ consumption that is typical of yeasts, which creates an anaerobic micro niche to permit the growth and biofilm formation of these strict anaerobic bacteria under aerobic conditions.^36^ Furthermore, lactic acid is the most preferred source of carbon for fungi under the hypoxic conditions created by *C. albicans*. Excluding metabolic interactions, *Candida* species also demonstrate positive physical interactions with bacteria. For example, co-aggregation promotes the growth of fungal cells in the biofilm core with bacteria around their periphery. Additionally, the *Treponema* flagellum forms a “bridge” between fungi and bacteria. With respect to chemical interactions, fungal ethanol secretion can enhance the growth and virulence of *Acinetobacter baumannii*. In contrast, bacteria may develop antibacterial tolerance by living under the protective fungal matrix umbrella.^37^ Through the rapid consumption of molecular oxygen, the rapid increase in the local pH, the provision of a physical scaffold for the adhesion of oral bacteria and the production of chemical factors that modulate oral bacteria, shifts in fungal communities may be a driving force for those that occur in bacterial communities. *Mycobacterium* infections have been shown to be associated with aspergillosis.^38^ The abundance of *Candida tropicalis* has been observed to be positively correlated with the presence of *Serratia marcescens* and *Escherichia coli*.^18^ Although fungi only constitute approximately 0.1% of the total microbial load in the oral cavity,^21^ at least 10% of the biovolume compensates for the presence of these microbes.

An ecological network is a representation of various biological relationships connected by pairwise links within an ecosystem.^39^ By analysing and then visualizing the spatial Pearson’s correlations between fungi and bacteria detected from saliva samples, an imbalanced microbial network was observed in patients with OLP. First, OLP patients, particularly those with erosive OLP, showed simpler co-occurrence patterns between the mycobiome and bacteriome, as evidenced by lower connectivity and higher modularity, suggesting that the fungal and bacterial nodes in the OLP networks were more sparsely connected. In addition, in the sub-networks, the correlation between Bacteroidetes and fungal species was increased, but the correlation between Firmicutes and fungal species was decreased in OLP, consistent with previous observations, such as the rapid consumption of molecular oxygen and the rapid increase in local pH. On the one hand, most Bacteroidetes (including *Prevotella* and *Porphyromonas*) are strictly anaerobic bacteria, which may be favoured by fungi at the expense of oxygen. Furthermore, *Bacteroides* excel at dominating the microbiota due to their ability to modulate surface polysaccharides in an effort to evade the host immune system.^40^ On the other hand, the consumption of lactic acid by fungi causes the environment to become less acidic, which may influence the growth of most Firmicutes members (such as *Lactobacillus* or *Streptococci*). Moreover, *Lactobacillus* sp. stimulate the mammalian host to induce antifungal immunity in the mucosal membrane.^21^ Additionally, as the most prevalent genus of the phylum Firmicutes, the abundance and networks of *Streptococci* were decreased in OLP patients, as was reported in our previous study^1^ and in a separate study.^41^ The alteration of such correlations indicates that active roles for the phyla Firmicutes and Bacteroidetes may be important for the severity and exacerbation of OLP. The opposite scenario has been observed with respect to obesity, inflammatory bowel diseases, and autism spectrum disorders, with increased Firmicutes and decreased Bacteroidetes observed. The phylum Firmicutes is enriched for genes encoding nutrient transporters, while the phylum *Bacteroidetes* enriched for genes linked to carbohydrate metabolism.^42^ However, our results are in agreement with those of other studies. Sam et al.^19^ observed an association between *Candida* and *Bacteroides*. Members of the genus *Bacteroides* are more abundant in individuals who consume a high protein diet, while the abundance of *Candida* is strongly associated with the recent consumption of carbohydrates. Thus, an increased connection between *Bacteroides* and fungi might contribute to OLP severity.

Emerging evidence suggests that the entire community of microbial residents influences the balance of immune responses, and microbial community dysbiosis may lead to deficient education of the host immune system followed by immune-mediated diseases.^40^ Furthermore, the expression of proinflammatory cytokines (e.g., IL-17 and IL-23) may be up-regulated by the presence of pathogens and the immunomodulatory components of biofilms (e.g., fungal glucans and bacterial lipopolysaccharides), resulting in tissue damage and lesions.^18,22,32^ In particular, IL-17, an inflammation-associated cytokine that reflects the immune dysregulation status, has emerged as a central player in the immunopathogenesis of OLP^15,16,43^ Previously, we analysed a potential association between the oral microbiome and IL-17 and IL-23 levels in the saliva of OLP patients.^1^ In this study, we further screened oral fungal genera that are potentially associated with disease severity and immune dysfunction of OLP. In total, 23 fungal genera were analysed, none of which were significantly associated with IL-23. A significant positive correlation was observed between IL-17 and 18 fungal genera, including *Dothiorella*, *Sympoventuria*, *Mycosphaerella*, and *Psathyrella*. In a previous study, the abundance of *Psathyrella* was significantly associated with Crohn’s disease, supporting the results of a previous study showing that IL-17 was essential for host defence against fungal infection.^44^ Notably, the genera *Bovista* and *Erysiphe* showed significantly positive correlations with clinical scores, suggesting their involvement in the aggravation of OLP. Thus, they were defined as keystone fungal genera^37^ that can modulate the host and the ecology in a manner that far outweighs their numerical representation in the community. Our results were consistent with those of a study by Wheeler et al.,^45^ who inoculated typically rare fungi (*Aspergilllus amstelodami*, *Epicoccum nigrum*, and *Wallemia sebi*) in mice and observed exaggerated immune responses, suggesting that these keystone fungi play important roles in immune homoeostasis. Despite a scarcity of data, the antifungal treatment of OLP patients has been shown to improve the clinical symptoms of OLP.^46^ Another study also described the involvement of fungi in the aggravation of inflammatory responses and the severity of gastrointestinal diseases.^12^ Based on the correlation between the myco-bacteriome and clinical parameters observed in this study and in a previous investigation,^43^ we suggest that the mycobiome may interact with commensal bacteria to augment the mucosal inflammatory response. In contrast, cytokines IL-17 had been shown to influence fungal composition and are important for protecting against infections caused by fungi (*C. albicans*, *Aspergillus fumigatus,* and *P. carinii*) on mucosal surfaces^6,15^ through the release of proinflammatory cytokines, chemokines, and anti-microbial peptides. A functional deficiency in the Th17 cell subset is associated with a dysbiotic state characterized by *Candida* overgrowth.^14^ Furthermore, signalling through the IL-17 receptor is crucial for protecting against candidiasis.^47^ The results of these studies demonstrated that IL-17 can play a central role on influencing the composition of core fungi, such as *Candida* and *Aspergillus*. Thus, it was supposed that keystone fungi can boost the host immunity (e.g., IL-17) and shape the core fungi composition through IL-17.

## Materials and methods

### Subject recruitment and sample collection

Subjects with reticular OLP (*n* = 17) and erosive OLP (*n* *=* 18), who were diagnosed according to the clinical classification and definition of the World Health Organization, together with 18 sex- and age-matched healthy controls were recruited from the West China Hospital of Stomatology, Sichuan University. Demographic information was obtained, and an oral examination was performed. A semiquantitative scoring system^23^ consistent with the site, area and presence of OLP lesions was used to assess the clinical scores and severity of OLP. All subjects included in this study had not received treatment for OLP for at least 2 months and were asked to avoid drinking or eating for 2 h before oral sampling. Those with other oral (e.g., periodontitis or dental caries) or systemic diseases were excluded. To reflect the structural changes of the entire microbiome in the oral cavity and adopt a painless approach, approximately 5 mL of spontaneous whole unstimulated saliva (WUS) was collected in a sterile DNA-free conical tube from each subject between 8:00 and 11:00 AM following standard techniques as described previously.^1^ All samples were carried to the laboratory on ice within 2 h and stored at −80 °C before further processing. The methods were performed in accordance with approved guidelines.

### Cytokine assay

IL-17 and IL-23 levels in the saliva were measured by ELISA as described previously.^43^

### DNA extraction

Genomic DNA was extracted from individual saliva samples using a Qiagen QIAamp^®^ DNA Mini Kit (Qiagen, Valencia, CA, USA) according to the manufacturer’s instructions as previously described.^48^ Briefly, after thawing on ice, aliquots were pelleted at 5 000 × *g* for 10 min and resuspended in 600 µL sorbitol buffer (1 mol·L^−1^ sorbitol, 100 mmol·L^−1^ EDTA, and 14 mmol· L^−1^ ß-mercaptoethanol). After incubating with 200 U lyticase at 30 °C for 30 min for cell lysis, protein digestion was achieved by adding Proteinase K and incubating the samples at 56 °C for 1.5 h. The DNA was bound to a spin column filter, washed with 96%–100% ethanol, and then was washed with the two buffers supplied by the kit. The bound DNA was eluted from the spin column filter with 200 µL of the supplied elution buffer. DNA quality was assessed by measuring the absorbance ratios using a Nano Drop-1000 Spectrophotometer (NanoDrop Technologies Inc., Wilmington, DE, USA). DNA samples with ratios of 1.8–2.0 (for A260/280 nm) and >1.8 (for A260/A230 nm) were likely to be free from contamination and were used for downstream experiments. Finally, the total DNA concentration was measured using a Pico-Green kit (Invitrogen, Carlsbad, CA, USA), and the extracts were frozen at −20 °C for further analysis.

### Illumina sequencing

The ITS2 region was amplified from the fungal DNA using the primers gITS7F (GTGARTCATCGARTCTTTG) and ITS4R (TCCTCCGCTTATTGATATGC), the product of which is expected to be 309 bp (not including the primers).^49^ A two-step phasing amplicon sequencing approach (PAS) was performed to avoid the amplification biases introduced by long barcoded PCR primers.^49,50^ Sample libraries for sequencing were prepared according to the 500-cycle v2 MiSeq Reagent Cartridge Preparation Guide (Illumina, San Diego, CA, USA) as described previously.^49^ Sequencing was performed for 251, 12 and 251 cycles for the forward, index and reverse reads, respectively, at the Institute for Environmental Genomics, University of Oklahoma (Norman, OK, USA). The barcoded 16S rRNA amplicon sequencing was performed using an Illumina MiSeq platform using the primers F515 (5′-GTGCCAGCMGCCGCGG-3′) and R806 (3′-TAATCTWTGGGVHCATCAG-5′) at the Institute for Environmental Genomics, University of Oklahoma (Norman, OK, USA). The amplicons obtained from all of the samples were then sequenced on an Illumina MiSeq platform.

### Data preprocessing, OTU clustering, and taxonomic classification

Data preprocessing and OTU clustering were performed as described previously.^51^ Only the reads with perfectly matched barcodes were extracted and used for further data analysis. Quality trimming of raw reads was carried out using the programme Btrim^52^ with an average quality score cutoff of 30 and a window size of 3. The paired-end reads were then joined using the programme pear^53^ with the default parameters. Further quality trimming and OTU clustering were carried out using the UPARSE pipeline. The joined reads were subjected to further quality control with a maximum expected error threshold of 0.5 and a length cutoff of 200. Qualifying reads were then dereplicated, sorted by size, and clustered into OTUs with 97% sequence identity. The OTU sequences were checked against the UNITE database, and potential chimeric sequences were removed. Finally, the qualifying reads were mapped to representative OTU sequences to calculate the relative abundance of each OTU. Taxonomic assignment for representative OTUs was carried out using the Ribosomal Database Project (RDP) classifier^54^ trained by the UNITE database. A confidence cutoff of 50% was used for taxonomic information assignments, and a random subsampling of 2 227 reads per sample was performed for further statistical analysis.

### Statistical analysis

The pre-processed data were further analysed using the following statistical methods. First, we used three different non-parametric multivariate analysis methods, including adonis (permutational multivariate analysis of variance using distance matrices), anosim (analysis of similarities), and multi-response permutation procedure (MRPP),^1^ as well as principle coordinate analysis (PCoA) to measure and visualize the overall differences in the fungal community structure between healthy and OLP individuals. Second, the fungal community diversity was assessed based on the Chao1 richness and Shannon diversity indices. Rarefaction analyses were performed using the programme Mothur^55^ by pooling samples within the same group. Student’s *t*-test was used to evaluate significant differences between healthy and OLP individuals, such as diversity indices and relative abundances of OTUs and taxonomic groups. Third, the Pearson correlation coefficient was used to construct bacterial–fungal co-occurrence patterns from the 16S rRNA gene and ITS amplicon data, which were also used for analyses of the association between fungi and clinical parameters. Bacterial and fungal OTUs present in more than eight samples were extracted and used for correlation calculations by clustering and visualizing using the MeV package.^56^ For better visualization, co-occurrence patterns with a Pearson correlation coefficient ≥0.6 and *P*-value ≤0.05 were extracted and plotted. Finally, OTU-level microbial co-occurrence networks were constructed and analysed. The random-matrix-theory-based approach in the MENA pipeline^57^ was used to construct the microbial co-occurrence networks. A Pearson correlation coefficient cutoff of 0.76 was determined by the random matrix theory approach by observing the transition point of the nearest-neighbour spacing distribution of eigenvalues from Gaussian to Poisson distributions, representing two universal extreme distributions. In such networks, OTUs were represented by network nodes, while correlations were transformed into the links between them. Sub-networks representing fungal–bacterial co-occurrence networks were subsequently constructed by extracting the first neighbours of the fungal OTUs. The co-occurrence networks were then visualized using Cytoscape 3.2.1.

## Supplementary information

supplemental tables and figures

## Data Availability

All the ITS2 and 16S rRNA sequences were deposited at NCBI under accession number SRP067603.
