# Supplementary material for: Salivary mycobiome dysbiosis and its potential impact on bacteriome shifts and host immunity in oral lichen planus
Source: Int J Oral Sci. 2019 Jul 2;11(2):13. doi: 10.1038/s41368-019-0045-2 (PMC6802619; doi:10.1038/s41368-019-0045-2)
Supplement: Supplementary file 1 — supplemental tables and figures [file 41368_2019_45_MOESM1_ESM.docx]

**Supplementary Materials**

**S1 Fig. Fungal structure comparison by PCOA among healthy controls, reticular OLP and erosive OLP.**  PCoA plot based on Bray-Curtis distances indicated no obvious separation.

H: healthy subjects; R: reticular OLP; E: erosive OLP.


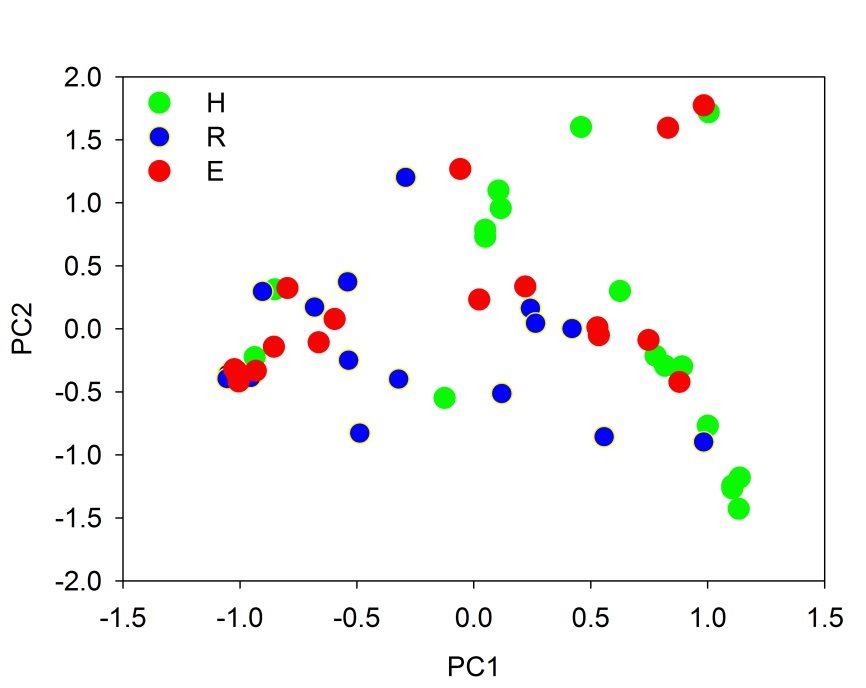


**S2 Fig. The core mycobiota of saliva samples among healthy controls, reticular OLP and erosive OLP.** The model included the genera (above 1% of relative abundance) and OTUs (above 0.3% of relative abundance) in healthy controls (green), reticular OLP (blue) and erosive OLP (red), which were found in at least 50% of subjects.(**a**): genus level; (**b**): OTU level.


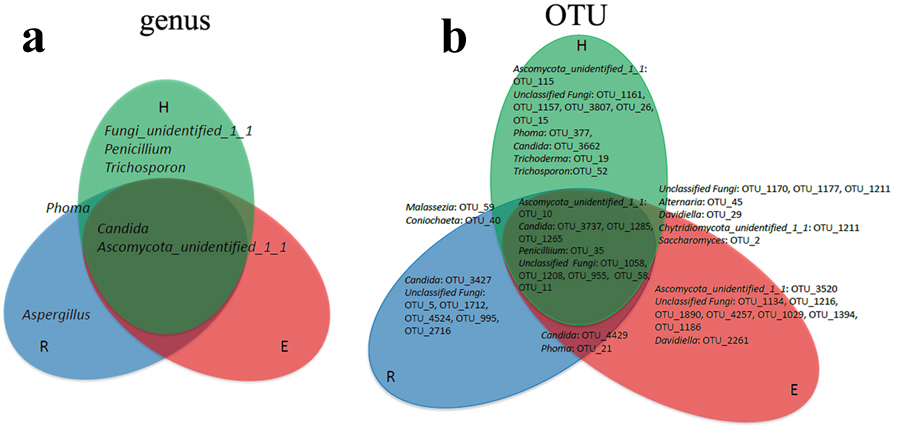


**S3 Fig. Network inferences for the complex fungal- bacterial relationships in OLP and healthy subjects.** Each node represents an OTU colored by the phylum-level phylotypes, and each edge represents a significant pairwise association. 9 bacterial phylum and 2 fungal (*Ascomycota*, *Basidiomycota)* were included. H: healthy subjects; R: reticular OLP; E: erosive OLP.

**
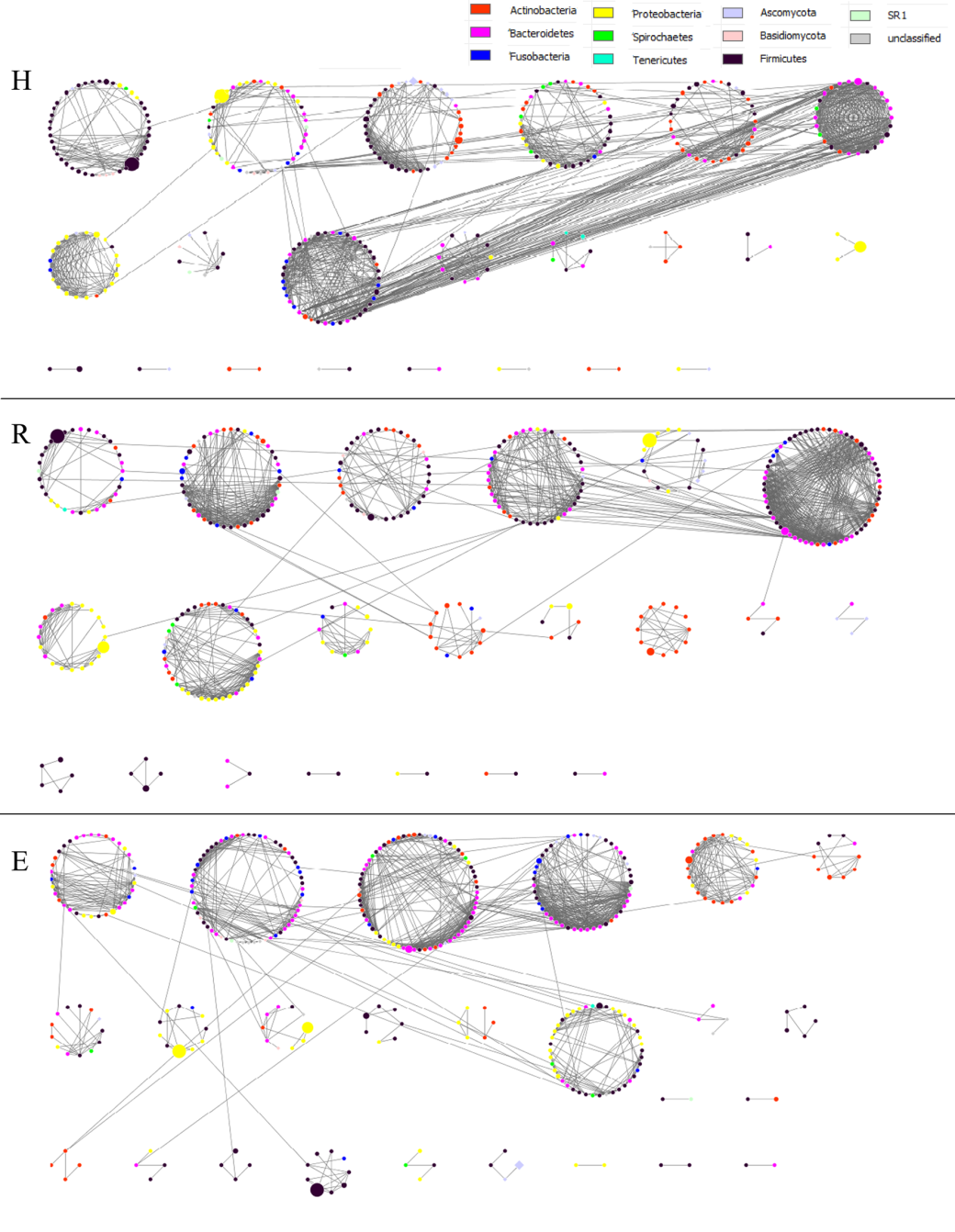
**

**S1 Table. Summary of mycobiome-bacteriome sequencing data among healthy and OLP patients**

|  | **Groups** | **#samples** | **#sequences per sample** | **#resampling efforts** | **#observed OTUs** | **Chao1 richness** | **Shannon index** | **Good’s coverage** |
| --- | --- | --- | --- | --- | --- | --- | --- | --- |
| Fungi | H | 18 | 25948±10182 | 2227 | 163±111 | 293±52 | 2.97±1.01 | 99.49±0.32 |
|  | R | 17 | 15636±6365 | 2227 | 117±63 | 180±26 | 2.42±0.97 | 99.69±0.27 |
|  | E | 18 | 13833±9742 | 2227 | 98±31 | 174±15 | 2.06±0.55 | 99.66±0.31 |
| Bacteria | H | 18 | 27094±20420 | 12997 | 459±112 | 748±247 | 3.60±0.45 | 99.86±0.3 |
|  | R | 18 | 62279±12523 | 12997 | 522±102 | 834±185 | 3.81±0.46 | 99.91±0.03 |
|  | E | 19 | 57528±16692 | 12997 | 585±160 | 944±259 | 3.86±0.48 | 99.74±0.6 |

Note: # means “numbers of”.

**S2 Table. Significant correlations between *Candida* and bacterial genera**

| ***Candida*** | **H** | **R** | **E** |
| --- | --- | --- | --- |
| *Abiotrophia* | -0.26178 | 0.170875 | -0.42148 |
| *Actinobacillus* | -0.08572 | 0.358064 | -0.12996 |
| *Aggregatibacter* | -0.2843 | 0.362404 | -0.14373 |
| *Bacteroides* |  |  | 0.112023 |
| *Brachymonas* | -0.07633 | -0.11342 | 0.328384 |
| *Bulleidia* |  | 0.038076 |  |
| *Capnocytophaga* | -0.37796 | -0.23747 | 0.37918 |
| *Catonella* | 0.02771 | 0.019852 | -0.35428 |
| *Cellulosimicrobium* | -0.33608 | -0.39456 | 0.271504 |
| *Dialister* | 0.031807 | 0.245904 | -0.63862 |
| *Eubacterium* | 0.250867 | -0.00878 | -0.06123 |
| *Gemella* | -0.23454 | -0.26895 | -0.05825 |
| *Haemophilus* | 0.235308 | 0.014587 | 0.277878 |
| *Johnsonella* | 0.40808 |  | -0.23228 |
| *Leptotrichia* | 0.154656 | -0.16702 | -0.36946 |
| *Moryella* | 0.280209 | 0.086017 | -0.03862 |
| *Peptococcus* | -0.13469 | 0.05674 | -0.56953 |
| *Peptostreptococcaceae_incertae_sedis* | -0.16565 | -0.08852 | -0.37154 |
| *Peptostreptococcus* | 0.053578 | -0.10123 | 0.000134 |
| *Planobacterium* | -0.25358 | -0.00821 | 0.282025 |
| *Porphyromonas* | -0.34648 | -0.06732 | -0.2592 |
| *Prevotella* | -0.18069 | -0.05006 | 0.026872 |
| *Rhizobium* | -0.39029 |  |  |
| *Schwartzia* |  |  | -0.30522 |
| *Solobacterium* | -0.06093 | -0.28639 | 0.007523 |
| *SR1_genera_incertae_sedis* | -0.20953 | 0.308775 | -0.593 |
| *Syntrophococcus* | -0.02797 | -0.04873 | -0.17608 |
| *Treponema* | -0.08413 | 0.111953 | -0.16836 |
| *Veillonella* | -0.02887 | 0.087615 | 0.262253 |

Note: Inversion of myco-bacteriome co-occurrence patterns from antagonization to co-prosperity in R group (green) and E group (red).

**S3 Table. Topological properties of co-occurrence networks of fungal communities in healthy subjects (H), reticular OLP (R) and erosive OLP (E)**

|  | **Total nodes** | **Total links** | **Average connectivity** | **Average path** | **Modularity** | **Centralization of degree** |
| --- | --- | --- | --- | --- | --- | --- |
| H | 336 | 1175 | 6.99 | 5.51 | 0.76 | 0.07 |
| R | 366 | 1241 | 6.78 | 6.05 | 0.77 | 0.06 |
| E | 383 | 1175 | 6.14 | 7.22 | 0.78 | 0.05 |
